# Impact of Minimally Manipulated Cell Therapy on Immune Responses in Radiation-Induced Skin Wound Healing

**DOI:** 10.3390/ijms26051994

**Published:** 2025-02-25

**Authors:** Victoria A. Shestakova, Ekaterina I. Smirnova, Dmitrii A. Atiakshin, Anastas A. Kisel, Sergey N. Koryakin, Evgeniy V. Litun, Vyacheslav O. Saburov, Grigory A. Demyashkin, Tatyana S. Lagoda, Anna O. Yakimova, Alexander E. Kabakov, Michael A. Ignatyuk, Elena M. Yatsenko, Dmitry A. Kudlay, Sergey A. Ivanov, Peter V. Shegay, Andrey D. Kaprin, Denis S. Baranovskii, Lyudmila N. Komarova, Ilya D. Klabukov

**Affiliations:** 1 National Medical Research Radiological Center of the Ministry of Health of Russian Federation, 249036 Obninsk, Russia; schestakova.vika2017@yandex.ru (V.A.S.);; 2 Department of Biotechnology, Obninsk Institute of Nuclear Power Engineering of the National Research Nuclear University MEPhI, 249034 Obninsk, Russia; 3 Scientific and Educational Resource Center “Innovative Technologies of Immunophenotyping, Digital Spatial Profiling and Ultrastructural Analysis”, Patrice Lumumba Peoples Friendship University of Russia (RUDN University), 117198 Moscow, Russia; 4 Institute for Translational Medicine and Biotechnology, Sechenov First Moscow State Medical University (Sechenov University), 119146 Moscow, Russia; 5 Immunology Department, Institute of Immunology FMBA of Russia, 115552 Moscow, Russia; 6 Department of Pharmacognosy and Industrial Pharmacy, Faculty of Fundamental Medicine, Lomonosov Moscow State University, 119992 Moscow, Russia; 7 University Hospital Basel, Basel University, 4001 Basel, Switzerland; 8 Research and Educational Resource Center for Cellular Technologies, Patrice Lumumba Peoples Friendship University of Russia (RUDN University), 117198 Moscow, Russia

**Keywords:** cell therapy, immune responses, ionizing radiation, mast cells, minimally manipulated cells, radiation injury, regenerative medicine, skin

## Abstract

The current treatment of radiation-induced skin wounds utilizes mainly conventional therapies, including topical steroids, creams, ointments, and hydrogel dressings, which do not take into account the immunologic changes that occur in the skin after radiation exposure. Therefore, it is relevant to consider alternative therapies and their impact on changes in the immune landscape of the skin. The aim of this study was to investigate the effect of allogeneic minimally manipulated keratinocytes and fibroblasts on rat skin repair and the development of immune responses. We found that the use of cell therapy compared to treatment with syntazone ointment and no treatment resulted in faster healing and a reduction in the size of radiation-induced skin wounds, area of inflammation, and edema. Additionally, in the group receiving the cell therapy application, there was an observed increase in the number of mast cells (MCs), activation of MC interaction with M2 macrophages, a reduction in the direct contact of MCs with the vascular bed, an increase in the content of collagen fibers due to the intensification of collagen fibrillogenesis, and a restoration of their histotopographic organization. Thus, the positive effect of cell therapy based on allogeneic minimally manipulated keratinocytes and fibroblasts on skin regeneration indicated that it can be used in clinical practice to improve the effectiveness of rehabilitation after radiation therapy.

## 1. Introduction

The treatment of non-healing radiation skin wounds, which are a common complication following radiotherapy or exposure to high levels of radiation, is a challenge in modern radiotherapy and radiation protection [1,2]. Radioprotectants are currently used to reduce the likelihood of radiation skin wounds. However, only a few radiotherapy drugs have been endorsed by the Food and Drug Administration [3], while others are approved only for emergency use [4]. Cell therapy for the treatment of radiation-induced skin wounds, particularly therapy with mesenchymal stem cells (MSCs) and exosomes derived from them, is being widely investigated. The favorable outcomes observed in the studies can be attributed primarily to the capacity of these cells to differentiate into a multitude of specialized cells within the body, modulate inflammatory processes, and stimulate tissue repair [5]. MSCs, with their anti-inflammatory, antifibrotic properties, immunomodulatory activity, superior regenerative potential, direct differentiation capability, and paracrine mechanisms, are a potential approach to mitigate and treat radiation-induced skin wounds [4,6,7]. However, challenges remain, including, as follows: the invasiveness of the medical procedure [8]; MSC heterogeneity [9,10]; low cell viability after transplantation [11]; poor homing and engraftment [10,11]; potential teratoma formation [9,12]; migration into tumors leading to tumor development and progression [13]; transmission of infectious diseases [9,14]; chronic disease progression [15]; genomic instability and immunoreactivity [16]; and the increased immunosuppressive properties of MSC secretome [17].

This dictates the need for Good Manufacturing Practice (GMP) facilities and comprehensive testing of cultured cell therapies; a considerable amount of time is thus required to obtain a license and appear on the market. In this regard, the development of methods based on manipulated cells that have not undergone significant manipulation is a particularly promising solution [18]. This technology enables the generation of fractions with therapeutic potential without the necessity of genotype and phenotype modification, thereby maintaining the intrinsic properties of the cells [19]. Keratinocytes and fibroblasts are commonly used, with autologous and allogeneic keratinocytes being the most studied [20]. Most likely, the greater use of keratinocytes and fibroblasts is due to the fact that these cells are able to produce classical hormones and neurotransmitters, thus regulating local skin homeostasis including in response to stress [21].

Keratinocytes are the main cell type in the epidermis responsible for forming the outer layer of the skin; they play a crucial role in wound healing by migrating to the wound site, proliferating and differentiating to rebuild the skin barrier [22]. During this process, keratinocytes secrete various growth factors and cytokines, including epidermal growth factor (EGF) and transforming growth factor-alpha (TGF-α), which stimulate cell migration and proliferation [23]. It was observed that keratinocytes synthesize, as follows: nitric oxide (NO), which regulates vascular permeability in the skin; ATP, which contributes to cutaneous sensory and inflammatory responses, and keratinocyte expression of receptors for melanocyte-stimulating hormone (MSH); and adrenocorticotropic hormone (ACTH) and β-endorphin, which provide regulation of keratinocyte proliferation and differentiation, as well as immune activity, wound healing, and cytoprotection [21]. Keratinocytes also secrete cytokines, chemokines, and antimicrobial peptides, facilitating communication with immune cells and promoting defense against pathogens [24]. Direct contact with fibroblasts stimulates keratinocyte proliferation and migration mediated by heparin-binding EGF-like growth factor (HB-EGF), interleukin-1 alpha (IL-1α), and transforming growth factor beta (TGF-β1) [25,26].

Unlike keratinocytes, fibroblasts are the primary cells of the dermis. Fibroblasts play a crucial role in wound healing by breaking down fibrin clots, creating new extracellular matrix (ECM) and collagen structures, and contracting the wound [27]. These cells interact with various ECM components, including collagens, fibrin, and proteoglycans, which influence fibroblast survival, migration, and metabolism [28]. While fibroblasts are essential for dermal repair, keratinocytes also contribute to skin homeostasis through active crosstalk with fibroblasts [29]. This interaction involves the production of cytokines, like IL-1α, by keratinocytes, which stimulate fibroblasts to produce keratinocyte growth factor (KGF) and metalloproteinases (MMPs). Understanding these complex cellular interactions and ECM components is crucial for developing potential therapies for fibrotic disorders and improving wound-healing outcomes.

Therefore, the aim of this study was to investigate the effect of allogeneic minimally manipulated keratinocytes and fibroblasts on rat skin repair and the development of immune responses. We hypothesize that the use of minimally manipulated cells as a novel therapeutic approach will result in qualitatively different immunologic responses in rat skin.

## 2. Results

### 2.1. Assessment of Viability of Isolated Cell Fractions According to the Developed Protocol

**Figure 1:**
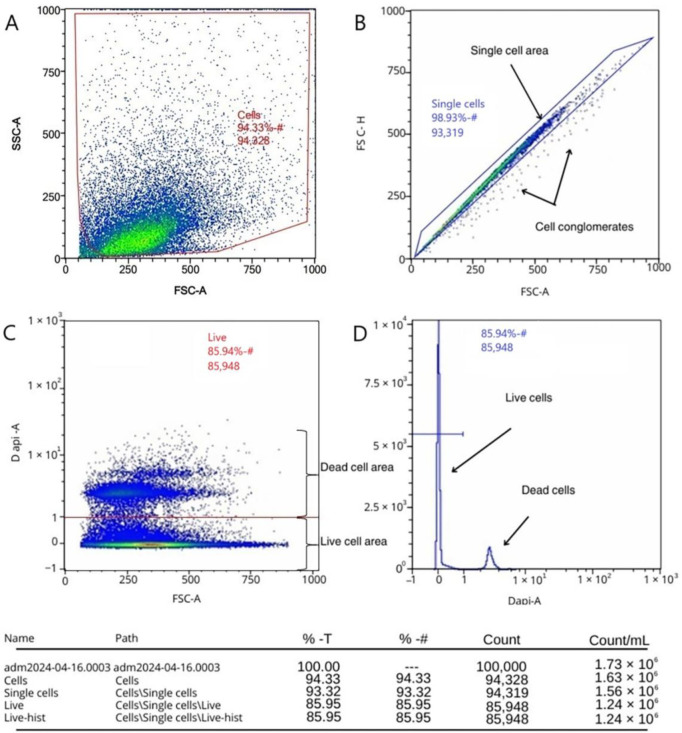
Assessment of viability of cell fractions using flow cytometry by staining with fluorescent dye DAPI: (**A**) exclusion of cellular debris; (**B**) exclusion of doublets and highlighting of the area of single cells; (**C**) live and dead cells; and (**D**) histogram of the distribution of live and dead cells.

The results of determining the viability of the isolated cell fractions using the developed protocol based on the flow cytometry method showed that, out of 100 thousand events of the investigated cell suspension, 5672 events fell on cell debris and cells outside the area of event registration (Figure 1A), which were not taken into account in further analyses. Of the 94,328 events that fell into the region of interest, 93,319 events (98.33%) fell into the “Single Cells” region (Figure 1B), indicating that the use of the developed protocol yields mostly single cells, with cell conglomerates accounting for slightly more than one percent of the events out of all events that fell into the region of interest. Direct analysis of the viability of cell fractions isolated using the developed protocol showed that the percentage of live single cells in the region of interest is 92.10%; in terms of all 100,000 events, this percentage is 85.95% (Figure 1C,D).

### 2.2. Cell Separation Based on Flow Cytometry and Sorting by Direct and Lateral Light Scattering

**Figure 2:**
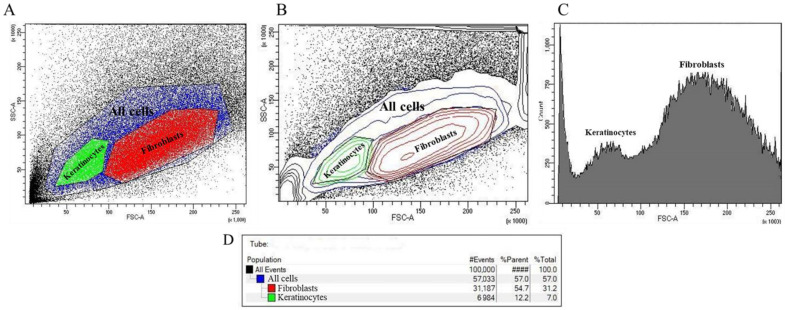
Results of flow cytometry by direct and lateral light scattering: (**A**) event density plot with selected polygonal regions “All cells”, “Keratinocytes” and “Fibroblasts”; (**B**) event density plot reflecting the nuclei of populations; (**C**) histogram of statistical distribution of cells by the number of events attributable to the regions “Keratinocytes” and “Fibroblasts”; and (**D**) distribution of events by populations.

Using BD FACS Aria III for the flow cytometry of cells enabled us to obtain two populations of cells differing in size and in the number of intracellular inclusions, as presented in Figure 2A. This separation is based on the displacement of the nuclei of the populations along the FSC-A and SSC-A axes, which can be seen in Figure 2B, and is supported by the data presented in the user manual [30]. Figure 2D shows that out of 100,000 events, 57,033 events fell into the “All cells” region of interest, which is 57.0%. Of these, 31,187 events (54.7%) fell into the “Fibroblasts” region, indicating that in the cell suspension under study, about 30% percent of the cells belonged to the fibroblast population, and the “Keratinocytes” region accounted for 6984 events out of all events, which corresponds to 12.2% of the events that fell into the region of interest and 7.0% of all events.

### 2.3. Morphometric Analysis

**Figure 3:**
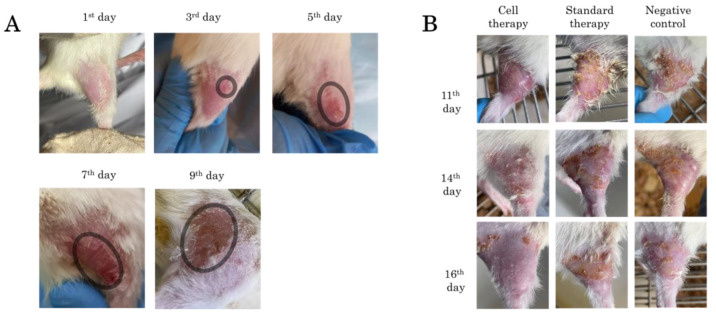
Skin appearance of rats after irradiation: (**A**) radial skin wounds development from day 1 to day 9, the area of radial lesion development is highlighted in the circle; and (**B**) radial skin wounds from day 11 to day 16 during application of Cell therapy, Standard therapy and Negative control.

Visible skin lesions were detected 3 days after irradiation. There was a general reddening of the irradiated area of the thigh skin with a small focus of inflammation. On the 5th day after irradiation, the redness persisted, with partial epidermis detachment and skin peeling in a small area of the thigh. Seven days after irradiation, the skin of animals in the control group was dry, reddish and scaly, with a papillomatous surface. On day 9, there was a resumption of fine hair growth along the edges of the irradiated area. General redness of the skin persisted; wet epidermitis developed, accompanied by tissue swelling (Figure 3A).

The results of changes in the irradiated skin area in the three experimental groups are presented in Figure 3B. On day 11, in the negative control group and in the treatment group, pronounced keratinization and peeling of the epidermis with scab formation was observed. Hyperemia, tissue edema and epilation of the irradiated area were preserved in all groups. On day 14, in the cell therapy group, edema subsided, hyperemia persisted in some places, and hair regrowth was observed. In the ointment-treated group and the negative control group, the scab was mainly located on the periphery of the wound, partially extending to the central area of the thigh; necrotic skin areas were visible with preserved hyperemia. On day 16, in the cell therapy group, there was almost complete recovery of the skin to its physiological state with a slight scab on the edges of the irradiated area, which is probably due to the fact that the cells were driven mainly to the central part of the thigh in the area of the main radial skin lesion. While the other two groups showed pronounced foci of tissue necrosis, hyperemia and scabs were preserved.

### 2.4. Histological Analysis

**Figure 4:**
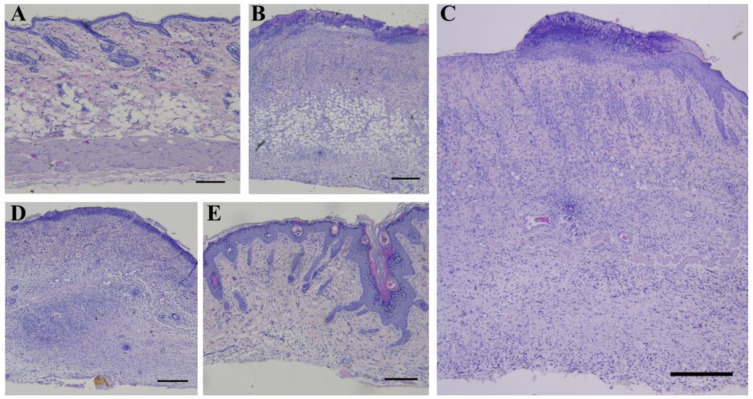
Hematoxylin and eosin staining: (**A**) non-irradiated and untreated native rat skin sample (control group); (**B**) skin sample of rats with radiation-induced skin wounds at the time of wet epidermitis development; (**C**) skin sample of rats with radiation-induced skin wounds without treatment at the end of the experiment (negative control group); (**D**) skin sample of rats treated with standard therapy; and (**E**) skin sample of rats treated with cell therapy. Scale bar 200 μm.

Hematoxylin and eosin staining showed that the native skin of rats in the control group consisted of well-defined epidermis, papillary and reticular layers of dermis, hypodermis, thin striated muscle layer (Panniculus carnosus), skin appendages and abundant hair follicles, which were also well visualized (Figure 4A), and the skin thickness was 828.88 ± 17.78 μm. The changes in the rat skin at the stage of wet epidermitis were accompanied by the development of radiation ulcer, necrotic ulceration, the appearance of homogeneous eosinophilic masses with marked granulocytic infiltration in the epidermis area, atrophy of sebaceous glands and hair follicles, edema, and marked lymphoplasmacytic infiltration; these were observed in the underlying dermis, which corresponds to granulation tissue. Such changes were observed in the hypodermis area (Figure 4B); the skin thickness was 1123.70 ± 8.54 μm. A similar transformation of skin tissue was observed in the negative control group of rats exposed to irradiation in the absence of treatment; however, in contrast to the wet epidermitis stage, there was a partial recovery of the epidermis (Figure 4C) with a skin thickness of 1140 ± 18.43 μm. When syntazone ointment was used as a treatment, there was preservation of the necrotic tissue, but in a smaller amount, and well-defined granulation tissue with vessels in the dermal layer were observed (Figure 4D). The skin thickness was close to the skin thickness during the wet epidermitis stage—1108.31 ± 24.41 μm. Skin staining of rats treated with cell therapy showed a reduction in inflammation after irradiation, decreasing the intensity of granulocytic infiltration in the epidermis and granulation tissue in the dermis, and restoration of hair follicles and sebaceous glands (Figure 4E). However, slight thickening of the spiny epidermis layer (acanthosis) and the presence of keratinized epidermis were observed, while the skin thickness was similar to that of native skin, and amounted to 825.63 ± 13.05 μm.

**Figure 5:**
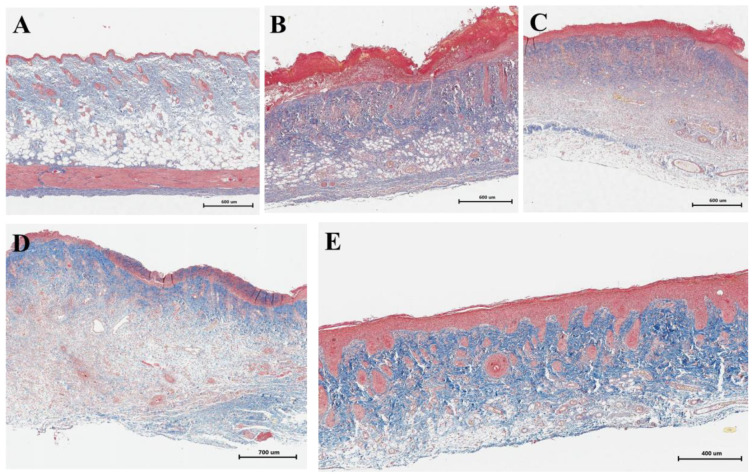
Picro Mallory staining: (**A**) non-irradiated and untreated native rat skin sample (control group); (**B**) skin sample of rats with radiation-induced skin wounds at the time of wet epidermitis development; (**C**) skin sample of rats with radiation-induced skin wounds without treatment at the end of the experiment (negative control group); (**D**) skin sample of rats treated with standard therapy; and (**E**) skin sample of rats treated with cell therapy.

Picro Mallory staining showed a relative decrease in collagen fiber content and an increase in cell concentration 9 days after irradiation at the stage of development of wet epidermitis (Figure 5B) and 16 days after irradiation in the negative control group (Figure 5C). Staining of the underlying dermis showed the presence of dense connective tissue and only a partial increase in collagen fiber content in the upper layers of the dermis (Figure 5D). In contrast, the density and intensity of collagen staining in the group of rats receiving cell therapy (Figure 5E) was physiologically significant and comparable to the skin of rats in the unirradiated control group (Figure 5A).

**Figure 6:**
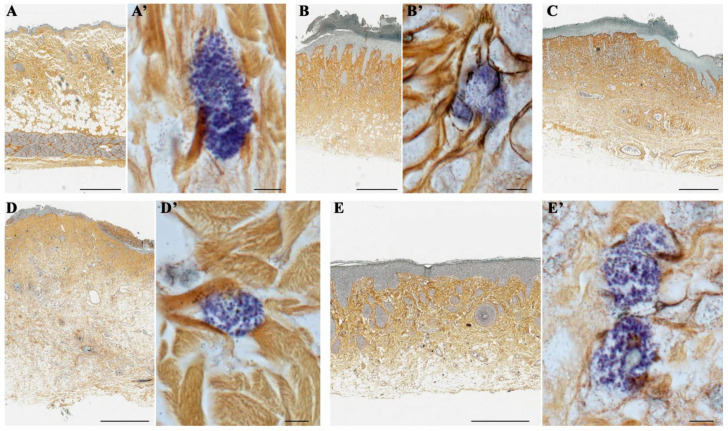
A combination of silver impregnation with toluidine blue staining: (**A**,**A’**) non-irradiated and untreated native rat skin sample (control group); (**B**,**B’**) skin sample of rats with radiation-induced skin wounds at the time of wet epidermitis development; (**C**) skin sample of rats with radiation-induced skin wounds without treatment at the end of the experiment (negative control group); (**D**,**D’**) skin sample of rats treated with standard therapy; and (**E**,**E’**) skin sample of rats treated with cell therapy. Scale bar: (**A**–**D**)—600 μm, (**E**)—400 μm, others—5 μm.

A combination of silver impregnation with toluidine blue staining, which is aimed directly at staining reticular fibers containing type III collagen and MCs, showed that in the control group, mast cells (MCs) were predominantly localized near collagen fibers (Figure 6A,A’). In the wet epidermitis stage, there is partial formation of thin reticular or pre-collagen fibers stained dark brown and small clusters of MCs are observed predominantly in the hypodermis (Figure 6B,B’). In the negative control group without treatment, thick and mature collagen fibers, stained yellow-brown, were mostly present and located throughout the dermis. Small accumulations of MCs were also observed, concentrated in the periwound skin areas, which was most likely due to their migration (Figure 6C). Application of syntazone ointment did not show effectiveness on the formation of new collagen fibers; however, the arrangement of the fibers became denser. The number of MCs was small, which is due to the peculiarities of the action of the main component of syntazone ointment—hydrocortisone acetate (Figure 6D,D’). In contrast, the application of cell therapy resulted in both the synthesis of new collagen fibers, their thickening and the formation of distinct areas in the dermis of the skin with particularly high MCs content (Figure 6E,E’).

In addition, it was noted that in the control group of animals without irradiation and treatment, collagen fibers were arranged chaotically (Figure 6A), whereas during the development of wet epidermitis, a weak linearization of collagen fibers was observed (Figure 6B); in the negative control group with irradiation but without treatment, a pronounced linearization of collagen fibers was observed (Figure 6C). The arrangement of collagen fibers in the group with syntazone ointment treatment and the group with cell therapy was characterized by the restoration of a chaotic arrangement of fibers (Figure 6D,E).

**Figure 7:**
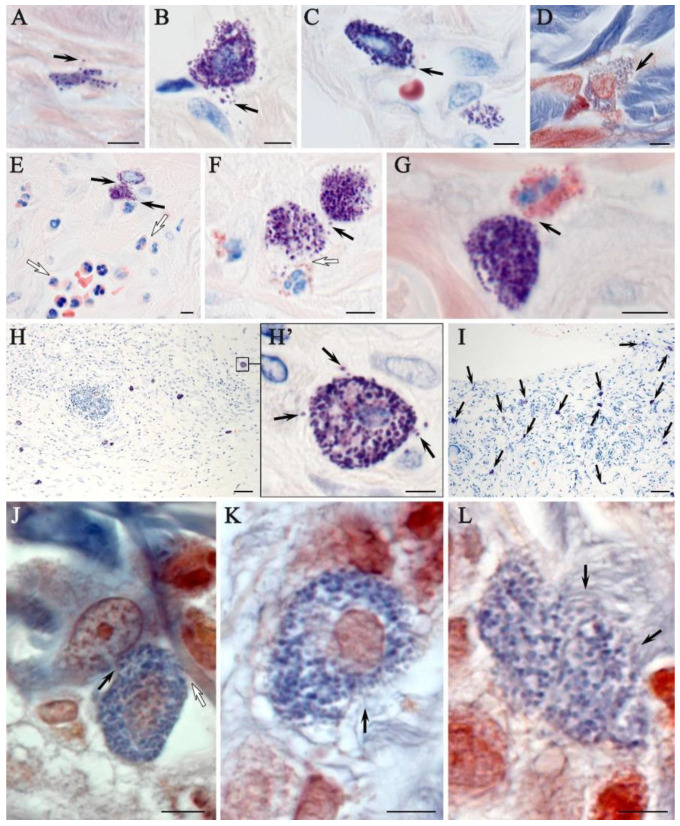
Histochemical equivalents of targeting mast cells to targets of the tissue microenvironment of the rat dermis of different experimental groups. Methods included (**A**–**I**) Giemsa staining and (**J**–**L**) picro Mallory staining protocols: (**A**–**D**) non-irradiated and untreated native rat skin sample (control group). The secretion of large granules with metachromasia (indicated by arrow) to thin collagen fibers of the papillary layer (**A**), fibroblast (**B**), capillary wall (**C**) and collagen fiber bundles of the reticular layer of the dermis (**D**) was determined; (**E**,**F**) skin sample of rats with radiation-induced skin wounds at the time of wet epidermitis development; (**E**) intercellular interaction of mast cells and neutrophil (arrow), against the background of accumulation of granular leukocytes in the venous section of the microcirculatory channel with signs of transendothelial migration to the tissue microenvironment of the dermis (white arrow); (**F**) interaction of mast cells with each other (arrow) and neutrophil granulocyte (white arrow); (**G**) skin sample of rats with radiation-induced skin wounds without treatment at the end of the experiment (negative control group). Co-localization of mast cell with eosinophil (arrow); (**H**) skin sample of rats treated with standard therapy; (**H’**) in a magnified fragment of (**H**), secretory granules in the extracellular matrix are detected (arrow). Skin sample of rats treated with cell therapy: (**I**) high mast cell content (indicated by arrow); (**J**) signs of targeted secretion of mast cell mediators to fibroblast (indicated by arrow) and collagen fiber (indicated by white arrow); and (**K**–**L**) loci with active collagen fibrillogenesis are identified in the pericellular space of mast cells (arrow). Scale bar: (**H**,**I**)—50 μm, others—5 μm.

Giemsa staining confirmed the presence of MCs, in similar areas identified by combined staining with toluidine blue and silver impregnation (Figure 7). Two subpopulations of MCs were observed in native rat skin, the smaller of which was located in the papillary layer of the dermis (Figure 7A); the larger ones were located in the reticular layer (Figure 7B). Smaller MCs were characterized by a low content of secretory granules. In deeper layers of the dermis, MCs were larger with a greater content of secretory granules and were often located near elements of the microcirculatory channel (Figure 7C). It was also observed that MCs predominantly localized near collagen fibers (Figure 7D). At the stage of wet epidermitis, high levels of neutrophils and eosinophils were detected in the vascular bed and elements of the microcirculatory channel, which, judging by morphological signs, were preparing for trans-endothelial migration into the connective tissue of the wounded skin area (Figure 7E). The interaction between MCs and neutrophils developed due to rare reactions occurring as a result of a very common phenomenon compared to native skin (Figure 7F). In the negative control group, increased MCs persisted along with continued interactions with neutrophilic and eosinophilic granulocytes (Figure 7G). The use of the ointment was accompanied by increased MCs content compared to that of native skin, but still remained small (Figure 7H). The group using cell therapy was characterized by the formation of some zones in the dermis of the skin with a particularly high content of MCs (Figure 7I). In these tissue loci, MCs actively interacted with other cellular and extracellular components of the tissue microenvironment, formed groups, and were characterized by high secretory activity. In particular, a pronounced interaction of MCs and fibroblasts was found, the co-localization of which is a sign of new collagen fiber formation (Figure 7J). This was confirmed by the observed signs of active involvement of MCs in collagen fibrillogenesis and remodeling of the extracellular matrix (Figure 7K,L).

### 2.5. Combined Immunohistochemical and Histochemical Staining and Monoplex and Multiplex Immunohistochemical Staining

**Figure 8:**
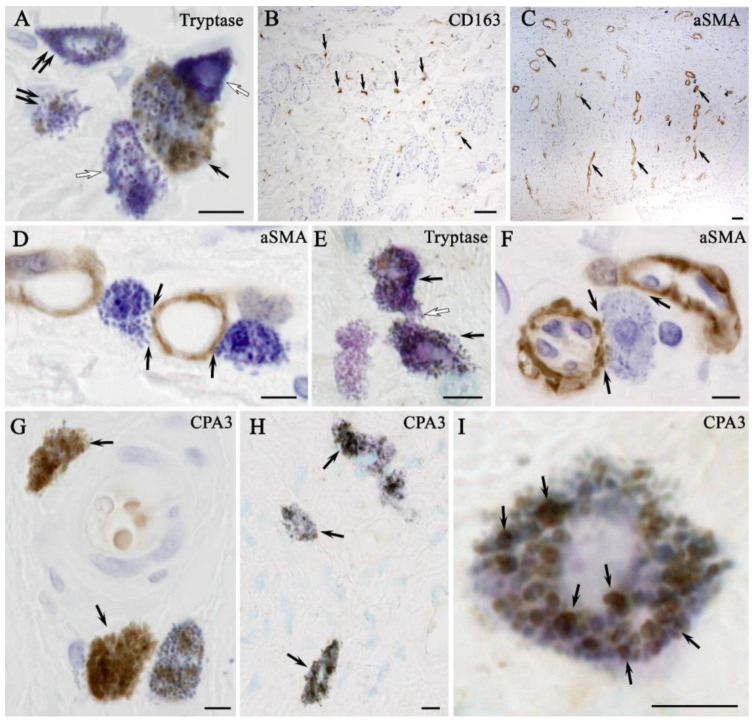
Expression and target secretion of mast cell tryptase in the skin of rats of different groups. Methods: Combined immunohistochemical and histochemical staining: (**A**,**B**) non-irradiated and untreated native rat skin sample (control group): (**A**) co-localization of mast cells with moderate (arrow), minimal (double arrow) and complete absence (white arrow) of tryptase expression; and (**B**) localization of type 2 macrophages in the connective tissue base of the skin (arrow); (**C**,**D**) skin samples of rats with radiation-induced skin wounds at the time of wet epidermitis development: (**C**) αSMA+ cells within the vasculature of the skin dermis (arrow); (**D**) targeted secretion of tryptase to structural components of the venule wall; (**E**) the skin sample of rats with radiation-induced skin wounds without treatment at the end of the experiment (negative control group), increased tryptase expression in most mast cells (arrow), intercellular interaction (white arrow); (**F**,**G**) skin samples of rats treated with standard therapy: (**F**) close co-localization of a degranulated mast cell with the microcirculatory channel of the skin dermis (arrow); and (**G**) mast cells are filled with granules with high tryptase content (arrow); (**H**,**I**) skin sample of rats treated with cell therapy: (**H**) most mast cells express tryptase; and (**I**) mast cell co-localized secretory granules with different levels of tryptase content, including high (arrow), peripheral localization of tryptase is identified in secretory granules. Scale bar: (**B**,**C**)—50 μm, others—5 μm.

The results of combined immunohistochemical and histochemical staining showed that the expression of tryptase and CPA3 by MCs was the lowest in native skin compared with the other experimental groups (Figure 8A). In addition, the dermis of the skin of intact animals had the lowest content of CD163 (Figure 8B). Electron irradiation led to an increase in the elements of the vascular bed. At the stage of wet epidermitis, the number of vessels increased and some of them had a vertical orientation to the wound surface (Figure 8C). The secretory activity of MCs to smooth myocytes, pericytes or endothelial cells within the vascular wall increased, increasing the degree of proinflammatory signaling to attract immunocompetent cells (Figure 8D). In the negative control group, there was an increase in the expression of both tryptase and CPA3 in MCs (Figure 8E). In the group using syntazone ointment, MCs actively interact with elements of the vascular bed. At the same time, some MCs exhausted their secretory resources as they lost most of their granules and, apparently, heparin (Figure 8F) (Supplementary Materials Figure S1). However, the content of specific proteases (tryptase and SRA3) remained commensurate compared to the no-treatment group, and significantly higher compared to the level in the control (Figure 8G). The specific interaction of MCs proteases with αSMA-positive cells was clearly visible in confocal microscopy and 3D imaging (Supplementary Materials Figure S2). The use of cell therapy resulted in the maintenance of high tryptase levels in the MC population. However, it should be noted that the formation of groups of MCs in similar histotopographic areas occurred, in which the level of tryptase expression was maximal. Similar data can be given regarding the specific MCs protease CPA3 (Figure 8H,I). This group also maintained a high degree of MCs degranulation, and interaction activity with α-SMA positive cells (Supplementary Materials Figure S2). At the same time, compared with the ointment group, MCs were found to be more frequently located around the vascular bed, at a paracrine distance from α-SMA positive cells; often, other cells were located between the MCs and the vessel. Despite this pattern, the MC population retained cells that actively interacted with the microcirculatory channel (Supplementary Materials Video S1).

**Figure 9:**
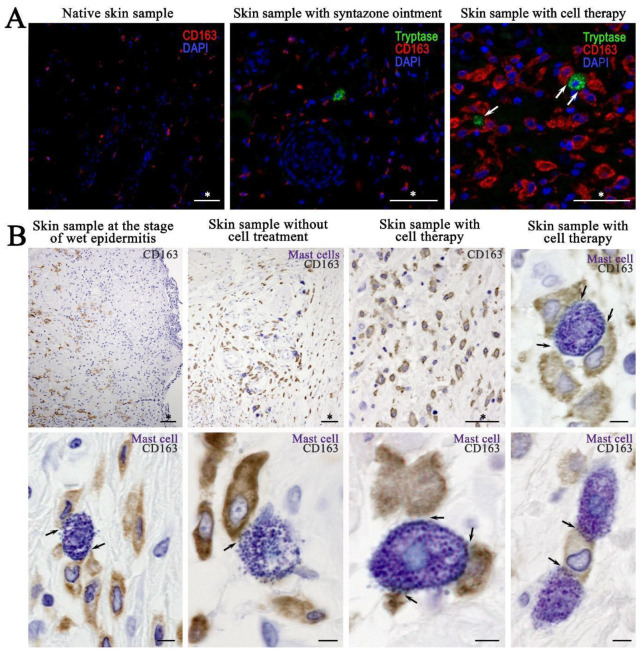
The content of M2-macrophages and their interaction with mast cells and their secretory granules: (**A**) monoplex and multiplex immunohistochemical staining; and (**B**) combined immunohistochemical (CD163 detection) and immunohistochemical (toluidine blue) staining. Arrow indicates mast cell-macrophage type 2 interaction loci. Scale bar: (marked by asterisk (*))—50 μm, others—5 μm.

Monoplex and multiplex immunohistochemical staining of the skin CD 68 и CD163 of intact animals (control group) showed the lowest content of M1- and M2-macrophages (CD68^+^ and CD163^+^ cells, respectively) (Figure 9A). Combined immunohistochemical and histochemical staining showed that irradiation led to an increase in the number of macrophages (both CD68^+^ and CD163^+^ cells), which was consistent with the change in the number of MCs. In particular, at the stage of wet epidermitis, an increase in the number of M1- and M2-macrophages was observed together with the frequency of contact with MCs (Figure 9B). Similarly, in the negative control group with and without irradiation treatment, the increased number of M1- and M2-macrophages, as well as a high frequency of their interaction with MCs, persisted (Figure 9B). The content of CD68^+^ and CD163^+^ cells seemed to increase in the group using syntazone ointment as a treatment compared with that of the negative control group (Figure 9A). Compared to the ointment use group, cell therapy application tended to increase the number of CD163+ cells in the skin, as well as more frequent co-localization with MCs (Figure 9A). This pattern was also clearly visible when histochemical detection of MCs by toluidine blue was combined with immunohistochemical staining for CD163^+^ cells. Most of the MCs were simultaneously in contact with several M2-macrophages. At the same time, sometimes several MCs interacted with a single macrophage by contacting it at different poles (Figure 9A). In monoplex and multiplex immunohistochemical staining, the orientation of MC secretory activity toward M2-macrophages was also clearly observed. Tryptase was secreted predominantly on CD163^+^ cells, interacting with appropriate receptors or penetrating into the cell interior, including the nucleus, presumably by exosomal transport (Supplementary Materials Video S2).

## 3. Discussion

Our preliminary results support the hypothesis that minimally manipulated allogeneic keratinocytes and fibroblasts can induce qualitatively different immunologic responses in irradiated rat skin. These changes appear to be a consequence of the active involvement of keratinocytes and fibroblasts in the process of skin regeneration. These results expand the frontiers of understanding the immunologic profile of the skin when using cell therapy to treat radiation-induced skin lesions and developing therapeutic approaches based on minimally manipulated cells.

Minimally manipulated keratinocytes and fibroblasts have previously been shown to have unique properties that modulate the local immune environment, promoting a favorable healing response [31,32]. However, most of the data concerned mesenchymal stem cells and their subtypes; there was a lack of information on the effects of minimally manipulated keratinocytes and fibroblasts on modulation of the immune environment. There is evidence that allogeneic keratinocytes and fibroblasts have shown promising results in full-thickness wound healing without evidence of immune rejection [33,34]. We compared morphologic and immunologic changes in rat skin in the absence of treatment, in the traditional treatment with syntazone ointment and in cell therapy with minimally manipulated cells and found that minimally manipulated cells produced the appearance of immunologic reactions in rat skin that were previously weakly expressed in other groups. These changes appear to be due to the ability of minimally manipulated cells to migrate to sites of injury, interact with resident immune cells, and facilitate the transition of macrophages from a pro-inflammatory M1 phenotype to a reparative M2 phenotype, which is essential for tissue repair and regeneration. It was found that minimally manipulated cells contribute to a reduction in inflammation, restoration of hair follicles and sebaceous glands; and the synthesis of new collagen fibers, their thickening and restoration of their chaotic organization after irradiation. It should be separately noted that cell therapy led to an increase in the number of MCs and their degranulation, activation of MCs interaction with M2-macrophages, and a reduction in the facts of direct contact of MCs with the vascular bed. Thus, minimally manipulated cell therapy leads to a decrease in proinflammatory signaling and migration of immunocompetent cells into the tissue microenvironment, which in turn accelerates the regenerative process due to high activity of the MCs in the remodeling of new collagen fibers, and reduces inflammation due to the direct contact of MCs with macrophages.

The skin is particularly susceptible to radiation because it is a constantly renewing organ due to the rapid multiplication and maturation of its constituent cells [35,36]. Radiation-induced skin wounds are therefore a serious problem in both radiotherapy and radiation accidents. This type of injury differs from traditional wounds in that the healing process is complex and the immunologic profile of irradiated skin differs significantly from that of acute traumatic, thermal, or chemical wounds, in which structural tissue damage occurs immediately or almost immediately [37]. The initial skin response to irradiation is characterized by an inflammatory reaction, which may manifest as erythema, edema, and, in severe cases, blisters and ulcers. This acute phase is driven by the release of proinflammatory cytokines and the recruitment of immune cells, such as neutrophils and macrophages, to the site of injury [38]. However, in contrast to normal wound healing, where the immune response is largely protective and promotes tissue repair, radiation-induced injuries often result in dysregulation of the inflammatory response [39]. Despite the skin’s ability to resist various stress factors and maintain local homeostasis through complex neuroendocrine mechanisms that play a crucial role in the skin’s response to trauma and healing, emphasizing the dynamic nature of the skin in regulating its microenvironment [21], neuroendocrine connections and mechanisms in the skin under irradiation have not yet been described. Therefore, the prolonged presence of inflammatory mediators can lead to a state of chronic inflammation [40]. As the inflammatory response progresses, the immune profile of irradiated skin changes. The initial influx of neutrophils is usually followed by a shift to macrophages, resulting in a predominance of proinflammatory signaling that prevents effective healing [41].

MCs play a crucial and specialized role in the process of skin regeneration, actively contributing to the regeneration of structural components of the connective tissue surrounding the wound [42,43]. They are activated immediately after skin damage and participate in all phases of the wound process [42]. This involvement is particularly evident when comparing the number of MCs in native skin versus those present in radiation-induced skin lesions. In these lesions, the density of MCs is markedly elevated, indicating a robust response to injury. MCs release a variety of mediators, including inflammatory mediators, proteases, and growth factors, which trigger a cascade of reactions and regulate the local immune response [42,44]. These mediators are important for recruiting other immune cells, such as macrophages, to the wound site [42,45]. In addition, the number of MCs expressing specific proteases, such as tryptase, was significantly higher in the treatment groups than in the skin of intact animals and in the negative control group. The increased expression of specific proteases in MCs further enhances their ability to remodel the extracellular matrix (ECM), facilitating the transition from inflammation to the proliferative phase of healing. MCs influence the behavior of fibroblasts, the primary cells responsible for collagen production, by secreting factors that stimulate fibroblast proliferation and differentiation [46]. This interaction is vital for the formation of a robust fibrous matrix that provides structural support for the new, regenerated tissue [47]. Moreover, MCs promote fibrillogenesis, the process by which collagen fibers are formed and organized into a functional ECM. These data support our findings on the synthesis of new collagen fibers, their compaction and the restoration of chaotic organization. In addition, it was found that MCs contribute to both arteriogenesis and angiogenesis by increasing blood flow, collateral diameter, and capillary formation in ischemic tissues [48]. They release angiogenic factors, such as vascular endothelial growth factor (VEGF) and fibroblast growth factor (FGF), which stimulate endothelial cell proliferation and migration [49]. This confirms our findings on the role of MCs attracted by cell therapy in modulating vascular tone, promoting angiogenesis, and enhancing vascular remodeling.

Another important component of successful skin repair is the interaction between MCs and macrophages by attracting macrophages to the site of injury [50,51]. Moreover, MCs can modulate macrophage polarization. By producing cytokines, such as IL-4, MCs promote macrophage polarization toward an anti-inflammatory phenotype [50]. It is most likely this mechanism that ensured the presence of macrophages II in the cell therapy group and the use of syntazone ointment. In this case, direct contact of MCs and macrophages coordinated the synthesis and degradation of extracellular matrix components, ensuring proper healing, restoration of skin architecture, and transition from the stage of inflammation to tissue repair.

Thus, our research points to the usefulness of minimally manipulated cell therapy in the search for immune modulation and skin repair after injury from beta radiation exposure. Enhancing both the acute inflammatory response and its sequential progression may make this therapeutic approach clinically applicable for the treatment of radiation injury. However, in order for this to be realized, more work needs to be conducted within this field, namely, in explaining the mechanisms involved and applying the findings clinically for patients exposed to radiation.

Although our study provides valuable insights, some limitations must be recognized. The use of a rat model, although useful for understanding basic immunologic and therapeutic processes, cannot fully reproduce the complexity of human skin responses. Furthermore, the utilization of a greater number of animals within each group has the potential to enhance the statistical power and generalizability of the findings. It is also important to note that a study involving a single acute skin exposure of 40 Gy cannot reflect the full range of radiation doses encountered in the context of clinical radiotherapy. A longer follow-up protocol would be beneficial in order to more fully evaluate the efficacy and outcomes of minimally manipulated cells in radiation-induced skin wounds.

## 4. Materials and Methods

### 4.1. Laboratory Animals

Female Wistar rats (n = 30; weight 190.8 ± 26.0 g; age 8–12 weeks) were divided into five groups (Table 1). All manipulations with laboratory animals were carried out in accordance with the recommendations and requirements of the Declaration of Helsinki and the approval of the Commission for Bioethical Control of the Keeping and Use of Laboratory Animals for Scientific Purposes of National Medical Research Radiological Centre of the Ministry of Health of the Russian Federation (protocol No. 1-N-00034 issued on 14 April 2023).

### 4.2. Ionizing Irradiation

Electron irradiation of the outer side of the thigh of the rats was performed using a 9 mm bolus to limit the irradiation area in the Department of Radiation Biophysics of the A. Tsyb Medical Radiological Research Center—branch of the FSBI “National Medical Research Radiological Centre” of the Ministry of Health of the Russian Federation, at the Novac-11 facility, with an electron energy of 10 MeV. The dose of single acute irradiation was 40 Gy, the duration of irradiation was 32.8 s. Fixation of the animals was performed using the cross method. Depilation of the irradiation area was performed using depilation cream (Stella, Russia).

The chosen radiation dose is the most commonly used dose in preclinical studies, equivalent to the doses used in clinical practice in the treatment of malignant skin tumors [52,53]. 

### 4.3. The Isolation of Minimally Manipulated Cell Fractions from Skin

The process of cell fraction isolation from skin consisted of several steps.

**Figure 10:**
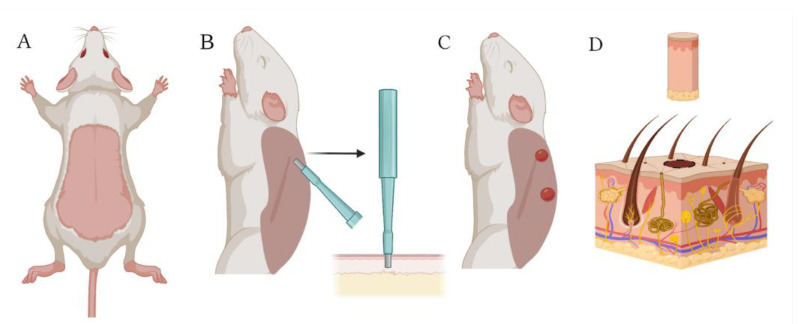
Collection of skin biomaterial using a biopsy punch: (**A**) dead rat with depilated skin on its back; (**B**) extraction of a skin biopsy specimen with a 7.5 mm biopsy punch; (**C**) appearance of wounds created on the rat’s back with the biopsy punch; and (**D**) skin biopsy specimen containing epidermal and dermal cells. Created with Biorender.com.

Initially, skin material was taken for cell isolation using a punch biopsy. For this purpose, rats from the donor group were first anesthetized by intraperitoneal injection of “Anestofol C” (active substance propofol (2,6-diisopropylphenol) (VIC, Vitebsk, Belarus) at a dose of 100 mg/kg body weight. Then, “Anestofol C” was injected intramuscularly at a dose of 32,000 mg/kg bodyweight to euthanize the animal. After this, depilation by plucking was performed and the shaved skin area was disinfected with 70% ethanol. The animal was then laid on its side, the dorsal skin was pulled away from the body, and a 7.5 mm biopsy punch (Apexmed International B.V., Amsterdam, The Netherlands) was used to obtain skin biopsy specimens, as shown in Figure 10. Similarly, using a new biopsy punch, the next pair of wounds were made 1–2 cm below the previous ones.

The obtained biopsy specimens were washed sequentially in 15 mL of sterile phosphate-buffered saline (PBS) and then in 10 mL of 70% ethanol for 30 s. The specimens were incubated twice for 5 min each in 15 mL of PBS supplemented with 1.5 mL of penicillin–streptomycin solution (Gibco, Grand Island, NY, USA) containing 10,000 U/mL penicillin and 10,000 μLl/mL streptomycin. After pretreatment, tissue samples were placed in sterile Petri dishes and thoroughly minced using a sterile scalpel or blade.

To isolate primary cell fractions, we used the developed protocol based on collagenase I (Gibco, USA) and dispase II (Sigma-Aldrich, St. Louis, MO, USA) at a concentration of 2.5 mg/mL and collagenase IV (Servicebio, Wuhan, China) at a concentration of 2 mg/mL, which were dissolved in a DMEM nutrient medium (PanEco, Gorki Leninskie, Russia). The obtained enzyme mixture was thoroughly mixed with homogenized shredded tissues and incubated in a CO_2_ incubator at 37 °C for 3 h with periodic stirring on a vortex. After 3 h, 1/5 volume of 0.25% trypsin (PanEco, Russia) was added to the enzyme mixture and incubated for another 30 min. After the time elapsed, the enzymatic cleavage process was stopped using a neutralizing medium (DMEM + 10% fetal bovine serum (FBS) + 1% penicillin/streptomycin) in a 1:1 ratio. The resulting solution was pipetted using a serological cell dissociation pipette and passed through a 100 μm cell filter into a new 50 mL conical tube to remove tissue debris. Then, 5 mL of the neutralizing medium was passed through the same filter to maximize cell yield. The resulting suspension of cells in the neutralizing medium was centrifuged at 500× *g* for 8 min at 20 °C. The supernatant was collected and 5 mL of the neutralizing medium was added, the cells were resuspended, the cell suspension with the neutralizing medium was passed through a 70 μm cell filter. The filter was washed with 5 mL of the neutralizing medium, and the sample was centrifuged again at 500× *g* for 8 min at 20 °C. The resulting cell suspension was lysed with Buffer LYS (SCI store, Moscow, Russia) to exclude skin erythrocytes from the cell suspension.

### 4.4. Assessment of Viability of Enzymatically Isolated Cell Fractions

Determination of the viability of isolated cell fractions, according to the developed protocol, was carried out using fluorescent dye DAPI (Thermo Fisher Scientific, Waltham, MA, USA) Initially, the number of cells in the obtained sample was counted using a Goryaev chamber. Then, 1–1.5 million cells were selected for analysis in a 5 mL assay tube (Falcon 12 × 75 mm) and 1–2 mL of PBS was added to the tube. The resulting cell suspension in PBS was centrifuged for 5 min at 400× *g* and the supernatant was removed. To the obtained cell fraction, 100 µL of DAPI diluted in sterile PBS at a concentration of 1 µg/mL was added, cells were stirred on a vortex for 10 s and placed in the refrigerator for 15 min. For staining after incubation, 1–2 mL of PBS was added and stirred on a vortex for 10 s. The cell suspension in the solution was re-centrifuged for 5 min at 400× *g*, the supernatant was removed, 500 µL of PBS was added, and the sample was analyzed on a MACSQuant Analyzer 16 flow cytometer (Miltenyi Biotec, Bergisch Gladbach, Germany).

### 4.5. Cell Separation Based on Flow Cytometry and Sorting by Direct and Lateral Light Scattering

Cell separation based on forward and side light scattering sorting was performed on a BD FACSAria III cell sorter (BD Biosciences, San Jose, CA, USA) equipped with FACSDiva software, version 9.0.1. For this purpose, a cell suspension at a concentration of 14.4 ± 0.21 million cells in 1 mL of phosphate-buffered saline were placed in an assay tube (PP, Falcon 12 × 75 mm) and then analyzed by flow cytometry on a BD FACS Aria III at 70 psi using an 85 μm nozzle and a flow rate of 1.0 to determine the populations. To obtain a uniform distribution of cells across populations, the optimum photomultiplier voltages for FSC (forward light scattering) were determined to be 265; for SSC (side scattering), optimal voltages were determined to be 375. After flow cytometry, cells were sorted according to all recommendations described in the BD FACSAria User Manual [54] into assay tubes soaked in fetal bovine serum (FBS) and filled with 1.5 mL of the DMEM nutrient medium supplemented with 10% FBS and 1% penicillin–streptomycin to preserve maximum cell viability.

### 4.6. Cell Therapy

For cell therapy, a cell suspension consisting of a 1:1 mixture of keratinocytes and fibroblasts was used, which was administered by injection. Before the procedure of cell suspension injection, the surface of the radiation-induced skin wounds was treated with disposable lint-free wipes with distilled water to remove surface contamination and then anesthetized by wiping the wound with disposable lint-free wipes moistened with 0.5% “Novocain” (active substance procaine hydrochloride) (“Grotex”, St. Petersburg, Russia). Rats were anesthetized by intracerebroventricular injection of “Anestofol C” at a dose of 100 mg/kg body weight. Cell injections were performed using needle applicators by multipoint injection into the basal membrane area. The volume of the injected cell suspension was 100 μL, the number of injected cells was 1 million, of which approximately 330 thousand were keratinocytes and 660 thousand were fibroblasts. The injection was performed once on the 9th day, at the moment of wet epidermitis development. The exposure time and duration of observation were 7 days. A suspension of keratinocytes and fibroblasts was used for cell therapy, administered by injection.

### 4.7. Treatment with Syntazone Ointment

Treatment with the syntazone ointment was carried out by superficial application of the ointment to the site of the radiation skin wound in a small amount with a spatula daily. Treatment was started on the 9th day after irradiation—during the period of wet epidermitis development. The duration of treatment and observation was 7 days, as in the case of the cell therapy treatment. During treatment, 5 animals were placed in 5 cages with one animal in each cage. Animals were dressed in homemade suits of dense fabric of an original cut to avoid ointment-licking by the animals.

### 4.8. Histological Examination

During the histologic examination, the following samples were used:

1. Skin samples of animals without radiation exposure (control group);
2. Skin samples of animals with radiation-induced skin wounds at the time of wet epidermitis development;
3. Skin samples of animals with radiation skin wounds without treatment at the end of the experiment (negative control group);
4. Skin samples of animals treated with standard therapy;
5. Skin samples of animals treated with cell therapy.

All samples were fixed in 4% formaldehyde for 72 h at room temperature, dehydrated in ethanol of increasing concentrations, and embedded in paraffin. Paraffin blocks were cut into serial slices of 2.5–5 μm thickness using a microtome (Thermo Fisher Scientific, CA, USA). The obtained slices were placed on uncoated slides and incubated at +56 °C for 30 min. The slides were deparaffinized and stained with hematoxylin and eosin (HE), picro Mallory and Giemsa staining according to the manufacturer’s protocol [55]. A combination of silver impregnation with toluidine blue staining was performed according to the protocol of Atiakshin D. et al. (2020) [56]. Histological images were acquired using a Leica DMI microscope (Leica Microsystems Gmbh, Wetzlar, Germany) and Aperio CS22 scanning microscope (Leica Microsystems Gmbh, Wetzlar, Germany), and analyzed using ImageScope 12.1 software.

### 4.9. Combined Immunohistochemical and Histochemical Staining

Combined immunohistochemical and histochemical staining was performed according to the principles outlined in the article by Atiakshin D. et al. (2021) [57]. The tryptase expression of the MCs was assessed using a combination of toluidine blue skin staining and immunohistochemical detection of tryptase. The combination of metachromatic detection of the MCs and immunohistochemical staining of α-smooth muscle actin (αSMA), macrophages type M1- (CD68^+^ cells), and M2- (CD163^+^ cells) not only demonstrated the content of α-positive cells, M1-, and M2-macrophages, but also their interaction with MCs. Stained sections were studied on a ZEISS Axio Imager.Z2 microscope (Carl Zeiss AG, Oberkochen, Germany).

### 4.10. Monoplex and Multiplex Immunohistochemical Staining

For the immunohistochemical analysis, deparaffinized slices were subjected to antigen unmasking process at 95 °C for 30 min in a specialized buffer, R-UNIVERSAL (Aptum Biologics Ltd., Southampton, UK). Blocking of endogenous Fc receptors prior to incubation with primary antibodies was not performed according to practice guidelines [58]. After blockade of endogenous peroxidase activity (if it was necessary), primary antibodies to tryptase, carboxypeptidase A3 (CPA3), αSMA, CD68, CD163 were applied at a concentration of 1 to 5 μg/mL and incubated overnight at +4 °C. Visualization of the primary antibodies in tissue structures was performed using secondary antibodies AmpliStain anti Mouse 1 Step (HRP) (SDT GmbH, Baesweiler, Germany) or AmpliStain anti Rabbit 1 Step (HRP) (SDT GmbH, Germany), conjugated with horseradish peroxidase and the DAB Peroxidase Substrate Kit detection system (Vector Labs, Newark, CA, USA).

For immunohistochemical staining with the simultaneous detection of multiple molecular targets, secondary antibodies conjugated to Alexa Fluor-488 fluorochromes (goat anti-rabbit IgG, #ab150077) or Cy3 (goat anti-mouse IgG, #ab97035) were used, with a final antibody concentration of 5 to 10 μg/mL PBS. Nuclei were contrasted using Mayer’s hematoxylin (BioVitrum, St. Petersburg, Russia) followed by the encapsulation of slices in permanent mounting medium, or with DAPI solution) for fluorescence labeling and using the VECTASHIELD Hard Set Mounting Medium (Vector Labs, Newark, CA, USA).

The stained slices were studied on a ZEISS Axio Imager.Z2 microscope. Some microphotographs were obtained using a Nikon D-Eclipse C1 Si confocal microscope (Nikon Corporation, Tokyo, Japan).

### 4.11. Images Processing and Statistical Analysis

Microphotographs of histological immunohistochemical and histochemical samples were processed using ImageJ software (version 1.54k) with the Fiji plugin and Aperio ImageScope 12.3. There was no quantification of the images and no statistical analysis of the values.

## 5. Conclusions

Our results showed that the use of minimally manipulated cell therapy for beta-radiation wounds results in accelerated healing of radiation-induced skin wounds and a significant reduction in the size of radiation-induced skin wounds, inflammation areas, and edema compared to conventional treatment or no treatment. The content of M2-macrophages (CD163^+^ cells) was found to be high compared to that of the other experimental groups; the interactions between MCs and CD163^+^ cells were activated. A higher activity of MCs in remodeling new collagen fibers was observed, which may contribute to more active regeneration. A decrease in the direct contact of MC with the vascular bed under the conditions of application of minimally manipulated cell therapy suggests a decrease in proinflammatory signaling and the migration of immunocompetent cells into the tissue microenvironment at this stage of regeneration.

## Figures and Tables

**Table 1:** Experimental design.

Group	n	Manipulations
I—donors	5	Skin biomaterial collection for cell isolation
II—control	5	Intact animals
III—negative control	5	Single local electron irradiation, dose—40 Gy, skin sampling at the stage of wet epidermitis development
IV—experimental	5	Single local electron irradiation, dose—40 Gy, absence of treatment
V—experimental	5	Single local electron irradiation, dose—40 Gy, treatment with standard therapy (Syntazone ointment)
VI—experimental	5	Single local electron irradiation, dose—40 Gy, cell therapy treatment

## Data Availability

Data are available on request.

## Supplementary Materials

The following supporting information can be downloaded at: https://www.mdpi.com/article/10.3390/ijms26051994/s1.
