# Supplementary material for: Impact of Minimally Manipulated Cell Therapy on Immune Responses in Radiation-Induced Skin Wound Healing
Source: Int J Mol Sci. 2025 Feb 25;26(5):1994. doi: 10.3390/ijms26051994 (PMC11900442; doi:10.3390/ijms26051994)
Supplement: Supplementary file 1 [file ijms-26-01994-s001.zip › Supplementary Materials.pdf]

Supplementary Materials

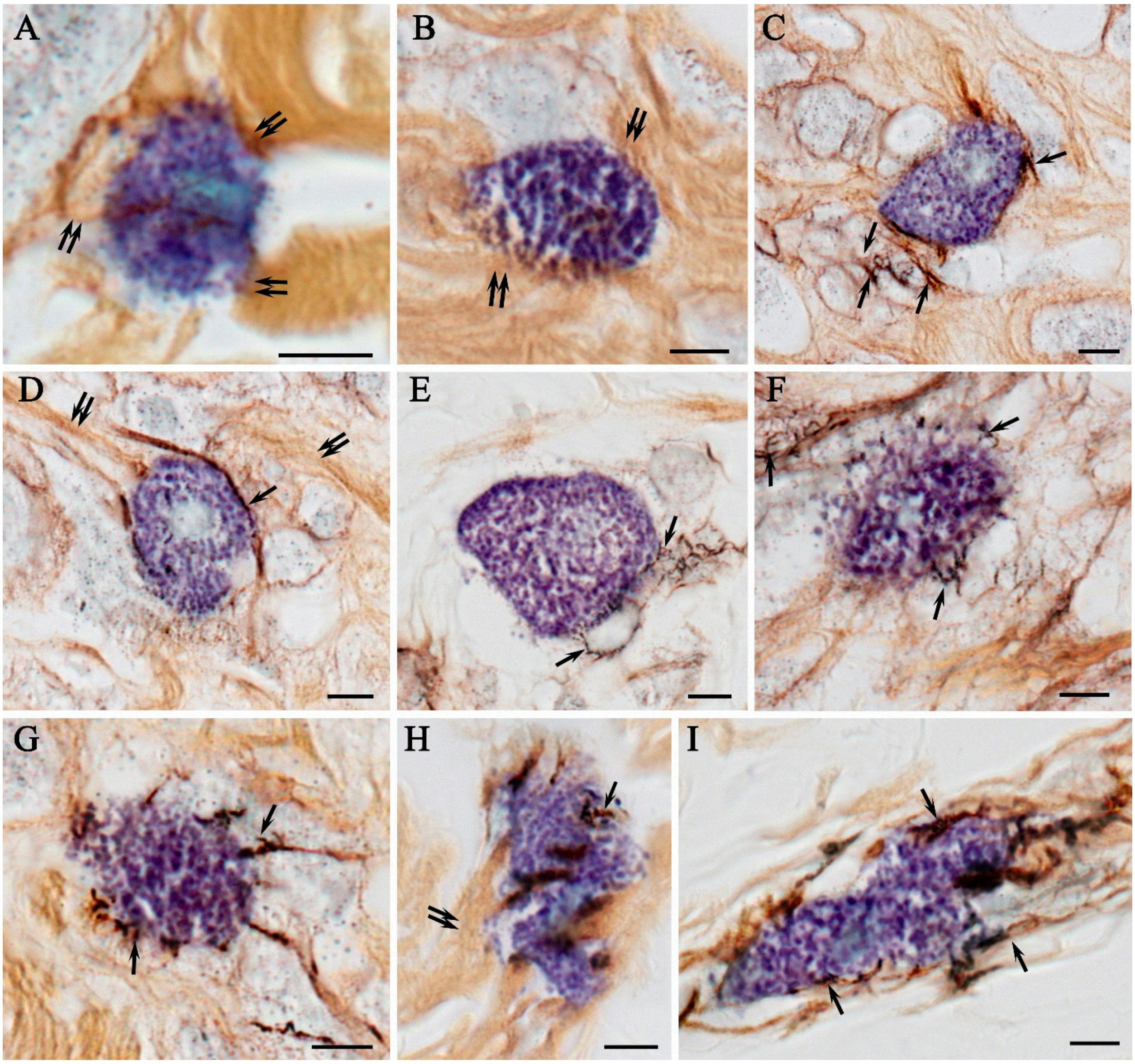

Figure S1. Participation of mast cells in remodeling of the extracellular matrix of rat skin dermis. (**A-B**) Non-irradiated and untreated native rat skin sample (control group). Mature collagen fibers predominate (double arrow). (**C**) Skin sample of rats with radiation-induced skin wounds at the time of wet epidermitis development. The microenvironment of mast cells reveals reticular fibers (arrow). (**D**) Skin sample of rats treated with standard therapy. (**E-F**) Skin sample of rats with radiation-induced skin wounds without treatment at the end of the experiment (negative control group). Mast cells co-localized with immature collagen fibers (arrow). (**G-I**) Skin sample of rats treated with cell therapy. Areas of active collagen fibrillogenesis are detected in the microenvironment of mast cells (arrow).

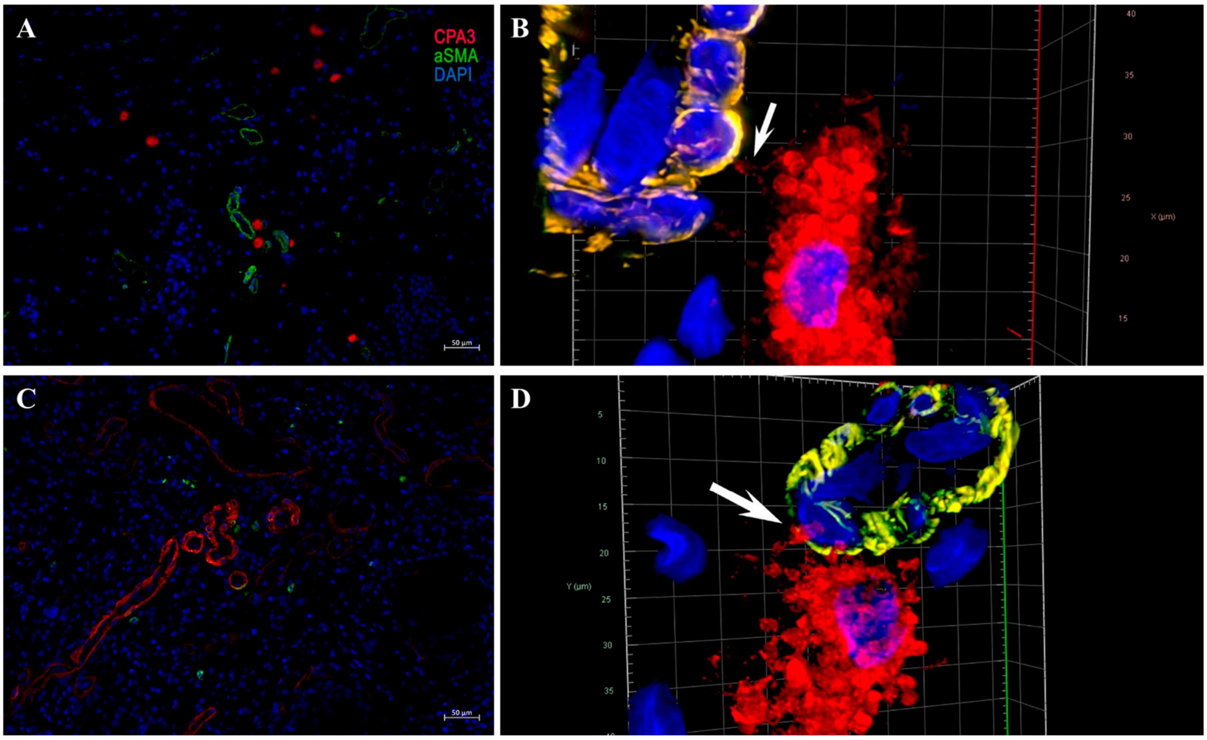

Figure S2. Monoplex and multiplex immunohistochemical staining. (**A**) Interaction of Mast Cells with elements of the vascular channel in the group treated with syntazone ointment. (**B**) Interaction of Mast Cell (MC) proteases with αSMA-positive cells in the group treated with syntazone ointment. (**C**) Interaction of MCs with elements of the vascular channel in the group of cell therapy. (**D**) Interaction of MC proteases with αSMA-positive cells in the group of cell therapy. Note: Staining with a mixture of Cy3 (red), Alexa Fluor 488 (green) and DAPI (blue) dyes. Arrows indicate interactions of mast cell proteases with αSMA-positive cells.

Video S1. Interaction of Mast Cell proteases with αSMA-positive cells in the group of cell therapy. Monoplex and multiplex immunohistochemical staining.

Video S2. Direction of Mast Cell secretory activity towards M2-macrophages. Monoplex and multiplex immunohistochemical staining.
